# Supplementary material for: Challenges and costs of donor screening for fecal microbiota transplantations
Source: PLoS One. 2022 Oct 20;17(10):e0276323. doi: 10.1371/journal.pone.0276323 (PMC9584411; doi:10.1371/journal.pone.0276323)
Supplement: S1 Table — a based on gender and/or CMV/EBV status; b PIMMS or FAIS study; c Donors of the TURN2-trial were additionally selected on a putatively favorable microbiota profile based on results from a previous TURN1 trial. Abbreviations: ESBL, extended spectrum beta-lactamase. (DOCX) [file pone.0276323.s001.docx]

| S1 Table. Demographics and reasons of exclusion of non-active donors. | | | | | |
| --- | --- | --- | --- | --- | --- |
| Donor | **Included study/studies** | **Age** | **BMI** | **Sexe** | **Reason of exclusion** |
| 1 | PIMMS | 41 | 24,7 | M | Antibiotic use |
| 2 | IMITHOT | 24 | 21,3 | F | ESBL-strain *Escherichia coli* |
| 3 | FAIS / TURN2 | 25 | 23,8 | F | No patient match ^a^, end of study ^b^ ; no favorable microbiota profile ^c^ |
| 4 | PIMMS | 28 | 24,8 | M | No patient match ^a^, end of study ^b^ |
| 5 | FAIS / TURN2 | 23 | 23,4 | F | No patient match ^a^, end of study ^b^ ; no favorable microbiota profile ^c^ |
| 6 | FAIS | 28 | 22,8 | M | No patient match ^a^, end of study ^b^ |
| 7 | TURN2 | 29 | 19,0 | F | No favorable microbiota profile ^c^ |
| 8 | TURN2 | 43 | 24,9 | F | No favorable microbiota profile ^c^ |
| 9 | TURN2 | 33 | 23,9 | F | No favorable microbiota profile ^c^ |
| 10 | TURN2 | 31 | 23,5 | F | No favorable microbiota profile ^c^ |
| 11 | FAIS / TURN2 | 26 | 20,1 | F | No patient match ^a^, end of study ^b^ ; no favorable microbiota profile ^c^ |
| 12 | IMITHOT/ TURN2 | 27 | 22,4 | M | No patient match ^a^, end of study ^b^ ; no favorable microbiota profile ^c^ |
| 13 | TURN2 | 29 | 23,9 | F | No favorable microbiota profile ^c^ |
| 14 | TURN2 | 30 | 19,7 | F | No favorable microbiota profile ^c^ |

^a^ based on gender and/or CMV/EBV status; ^b^ PIMMS or FAIS study; ^c^ Donors of the TURN2-trial were additionally
selected on a putatively favorable microbiota profile based on results from a previous TURN1 trial.

Abbreviations: ESBL, extended spectrum beta-lactamase.
