# Supplementary material for: Challenges and costs of donor screening for fecal microbiota transplantations
Source: PLoS One. 2022 Oct 20;17(10):e0276323. doi: 10.1371/journal.pone.0276323 (PMC9584411; doi:10.1371/journal.pone.0276323)
Supplement: S2 Table — a Determined microscopically by an experienced laboratory analyst [26]; b PIMMS or FAIS study; c based on gender or CMV/EBV status. Abbreviations: CBC, complete blood count; CRP, c-reactive protein; DFT, dual feces test; ESBL, extended spectrum beta-lactamase; NA, not applicable; STEC, shiga toxin-producing Escherichia coli; MDROs, multidrug resistant organisms; SARS-CoV-2, severe acute respiratory syndrome coronavirus 2. (DOCX) [file pone.0276323.s002.docx]

| S2 Table. Demographics, specifications of screening, and reasons of exclusion of active donors. | | | | | | | | | | | |
| --- | --- | --- | --- | --- | --- | --- | --- | --- | --- | --- | --- |
| Donor | **Included study/studies** | **Age** | **BMI** | **Sexe** | **Donations** | **Full screenings** | **60 days screenings** | **Additional screenings** | **Transient positive tests** | **Transient pathogens** | **Reason of exclusion** |
| 1 | IMITHOT | 34 | 19.6 | F | 6 | 2 | 3 | 0 | 0 | NA | Antibiotic use |
| 2 | FAIS | 27 | 21.8 | F | 12 | 5 | 3 | 5 (DFTs) | 2 | *Blastocystis* spp.  (‘rare’ or ‘few’) ^a^ | *Blastocystis* spp. (‘moderate’ or ‘many’) ^a^ |
| 3 | FAIS / TURN2 | 27 | 20.4 | F | 5 | 1 | 0 | 1 (serum CBC, CRP) | 0 | NA | *Blastocystis* spp. |
| 4 | TURN2 | 28 | 20.2 | F | 21 | 4 | 3 | 0 | 0 | NA | Changed occupation to health care worker |
| 5 | TURN2 | 29 | 25.0 | M | 19 | 3 | 3 | 5 (feces viral) | 2 | Enterovirus | Changed occupation to health care worker |
| 6 | IMITHOT / FAIS | 25 | 20.2 | M | 12 | 3 | 5 | 0 | 0 | NA | COVID-19 measures |
| 7 | TURN2 | 22 | 24.7 | F | 18 | 4 | 2 | 6 (DFTs) | 0 | NA | *Dientamoeba* *fragilis* |
| 8 | FAIS | 29 | 23.9 | M | 2 | 2 | 0 | 0 | 0 | NA | *Dientamoeba* *fragilis* + *Blastocystis* spp. |
| 9 | PIMMS | 29 | 23.7 | M | 9 | 2 | 2 | 0 | 0 | NA | End of study ^b^ |
| 10 | PIMMS | 33 | 20.6 | M | 3 | 2 | 1 | 1 (feces bacterial) | 1 | Yersinia enterocolitica | End of study ^b^ |
| 11 | IMITHOT / FAIS | 38 | 24.4 | F | 2 | 2 | 1 | 0 | 0 | NA | End of study ^b^ |
| 12 | TURN2 | 51 | 22.6 | F | 7 | 2 | 3 | 3 (MDROs) | 0 | NA | ESBL-strain *Escherichia coli* |
| 13 | PIMMS / TURN2 | 45 | 21.1 | M | 18 | 4 | 3 | 0 | 0 | NA | NA (active donor) |
| 14 | IIMTHOT / FAIS | 23 | 23 | M | 13 | 3 | 4 | 1 (SARS-CoV-2) | 0 | NA | NA (active donor) |
| 15 | TURN2 / IMITHOT / FAIS | 25 | 24.0 | F | 28 | 3 | 5 | 5 (feces viral) | 5 | Noro-, Sapo-, Enterovirus | NA (active donor) |
| 16 | TURN2 | 21 | 24.2 | M | 16 | 2 | 0 | 0 | 0 | NA | NA (active donor) |
| 17 | IMITHOT | 23 | 21.7 | F | 6 | 1 | 1 | 0 | 0 | NA | NA (active donor) |
| 18 | TURN2 / IMITHOT / FAIS | 27 | 19.0 | F | 28 | 4 | 3 | 1 (SARS-CoV-2) | 0 | 0 | No patient match ^c^ |
| 19 | FAIS | 31 | 21.0 | F | 2 | 1 | 0 | 0 | 0 | NA | No patient match ^c^ |
| 20 | IMITHOT | 22 | 21.6 | M | 3 | 1 | 3 | 3 | 0 | NA | No patient match ^c^ |
| 21 | TURN2 | 47 | 25.4 | M | 48 | 5 | 5 | 0 | 0 | NA | Personal circumstances |
| 22 | IMITHOT / PIMMS FAIS | 28 | 25.3 | F | 27 | 1 | 1 | 0 | 0 | NA | Rehousing |
| 23 | TURN2 | 26 | 18.9 | F | 14 | 3 | 4 | 3 (feces viral, STEC, MDROs) | 1 | Enterovirus | STEC + ESBL-strain *Escherichia coli* |
| 24 | FAIS / PIMMS | 25 | 20.7 | F | 6 | 2 | 1 | 0 | 0 | NA | Travel |

^a^ Determined microscopically by an experienced laboratory analyst.^38^; ^b^ PIMMS or FAIS study; ^c^ based on gender or CMV/EBV status.

Abbreviations: CBC, complete blood count; CRP, c-reactive protein; DFT, dual feces test; ESBL, extended spectrum beta-lactamase; NA, not applicable;

STEC, shiga toxin-producing Escherichia coli; MDROs, multidrug resistant organisms; SARS-CoV-2, severe acute respiratory syndrome coronavirus 2.
