# Supplementary material for: Challenges and costs of donor screening for fecal microbiota transplantations
Source: PLoS One. 2022 Oct 20;17(10):e0276323. doi: 10.1371/journal.pone.0276323 (PMC9584411; doi:10.1371/journal.pone.0276323)
Supplement: Attachment — Submitted filename: Response_to_Reviewers_23AUG2022.docx [file pone.0276323.s003.docx]

Franck Carbonero, PhD

Editorial board PLOS ONE

Amsterdam, the Netherlands, July 26th, 2022

Re:

Manuscript title:	“Challenges and costs of donor screening for fecal microbiota transplantations”

Authors:	Mèlanie Bénard, Clara Bruijn, Aline Fenneman, Koen Wortelboer, Judith Zeevenhoven, Bente Rethans, Hilde Herrema, Tom van Gool, Max Nieuwdorp, Marc Benninga, Cyriel Ponsioen

Manuscript ID:	PONE-D-22-15731

Dear Dr. Carbonero,

Thank you very much for considering our paper entitled: “Challenges and costs of donor screening for fecal microbiota transplantations” for publication. We would like to thank the reviewers for their valuable comments, which we addressed and hope has improved the manuscript.

First, we would like to emphasize that the funders (ZONMW) had no role in study design, data collection and analysis, decision to publish, or preparation of the manuscript.

Second, we would like to respond to the question if PloS One is the appropriate outlet for our manuscript. We chose to submit to PloS one because the scope of the journal as cited on the http://journal.plos.org website mentions that Plos One publishes submissions on methods and protocols. Over the past couple of years the rising number of registered clinical FMT trials worldwide highlights the sharply increasing interest in FMT. However, this field is quite new and there are no uniform protocols. In our center, we were the first to ever conduct a placebo-controlled, randomized clinical trial with FMT (van Nood et al. NEJM, 2013) for refractory *Clostridioides difficile* infection. Since then, we have built on our experience with FMT in a research and clinical setting. With this manuscript we aim to share our longstanding experience and provide tools from a practical perspective to clinicians, investigators, as well as institutions who wish to set up or improve an FMT service for clinical use and/or research. For these reasons, we believe that our manuscript is relevant and well-suited for publication in your journal. Also, in this respect we fully agree with the introductory notes of reviewer #2.

In a stepwise fashion, we numbered consecutively all comments and queries made by the reviewers and have addressed these point by point. All changes in the revised version of the manuscript are marked with the use of track changes. Our detailed point-to-point response to the comments is provided below.

We hope that the revised version of the manuscript is now suitable for publication in PloS One.

We look forward to hearing from you.

Best regards,

On behalf of the co-authors,

Cyriel Y. Ponsioen MD, PhD

Department of Gastroenterology & Hepatology

Amsterdam University Medical Centers

Amsterdam, The Netherlands

c.y.ponsioen@amsterdamumc.nl

The Netherlands

Tel: +31205662906

REVISION CHECKLIST

√ Please ensure that your manuscript meets PLOS ONE's style requirements, including those for file naming.

√ Please state what role the funders took in the study.  If the funders had no role, please state: ""The funders had no role in study design, data collection and analysis, decision to publish, or preparation of the manuscript."" Please include this amended Role of Funder statement in your cover letter; we will change the online submission form on your behalf.

√ Please include captions for your Supporting Information files at the end of your manuscript, and update any in-text citations to match accordingly.

REVIEWERS COMMENTS

Reviewer: 1

Response to the reviewers’ queries:

1. The statistical approach was primarily descriptive as there are no real comparisons being made. The overall 10% participation rate (38/393) is not very convincing from a statistical perspective. How relevant or generalizable the statistics presented are to a realistic screening cost attempt is doubtful. The investigators made no attempt to determine the true precision (using appropriate statistical sampling techniques) of any subject data based on this small sample.

With the current manuscript we aim to provide tools from a practical perspective to clinicians, investigators, as well as institutions who wish to set up or improve an FMT service for clinical use and/or research. Moreover, as our study included data regarding recruitment and selection procedures of healthy fecal donors from four different clinical FMT trials, we believe we created a relatively large cohort. By reporting the average costs associated with our donor screening program in our manuscript we provide an estimate for clinicians thinking of establishing a pool of healthy stool donors for FMT research. The generalizability of the presented screening costs are discussed on page 20-21. Due to the nature of the study aims, formal statistics are outside the scope of the manuscript.

2. Another deficit is that there was no effort to compare the subject characteristics seen in the various tables and supplemental tables in the active and non-active donor groups.

Main reason for donors to become non-active was the absence of a favorable microbiota profile (TURN2-trial). As described in the method section on page 7 a favorable microbiota profile is based on results from a previous TURN1 trial and include high alpha-diversity and high predicted butyrate production. Differences in subject characteristics with regard to the presence or absence of a favorable microbiota profile are outside the scope of the current study and will be published elsewhere within the context of the TURN2 trial. Hence, in this study, our aim was not to compare characteristics of active versus non-active donors.

3. The manuscript has to be edited for typos. On line 227 of the text the investigators state that,” Demographic characteristics of the active donors, details on (re)screenings, and reasons for later exclusion are listed in Supplementary Table S2". The active donor data is on Table S1.

We adjusted the titles of S1 Table and S2 accordingly.

Reviewer: 2

Benard et al. describe their experience in qualifying and sustaining stool donors for FMT trials for IBS, autoimmunity, obesity, and ulcerative colitis. They quantify the donor-associated costs within their specific protocol. Reporting this experience is valuable for several audiences: academic centers thinking about establishing their own program, commercial developers of microbiota-based therapeutics, and regulators.

Perceptions of how easy or hard it is to run a donor program are varied and contradictory. Some assume that such a program should be easy and inexpensive because stool is widely available. Others believe that procuring stool as raw material for therapeutics is fraught with insurmountable dangers and will ultimately be looked upon as a transient period in medical history forced by desperation.

It is important to note that this is still a very young field. Little is known about the dangers lurking in the stool of asymptomatic and seemingly healthy individuals. Experts often base their opinions on minimal evidence, which can be entirely hypothetical or anecdotal. There is often a somewhat flippant attitude to add to the lists of screening tests, which can then be accepted by regulators as settled knowledge and written in stone without adequate evaluation of true risks and benefits. There is the other side, common among many patients and do-it-yourself advocates, that FMT is entirely innocuous and free of dangers. These attitudes should be carefully considered in this manuscript.

Overall, I think the manuscript is very well written, and the following are my comments that I hope will be addressed:

Response to the reviewers’ queries:

1. The exclusion rate of donors based on findings of D. fragilis and Blastocystis spp. is striking. However, as the authors note, these ‘parasites’ are more likely to be innocuous commensals and may even be mutualist as the carriers may have lesser prevalence of GI symptoms and their microbiota may have more favorable characteristics (e.g., bacterial diversity, butyrate production). I think the modified protocol presented in the discussion is reasonable, although difficult to implement given subjectivity intrinsic to ‘high’ or ‘low’ abundance as determined by a laboratory technician. An RCT with D. fragilis/Blastocystis-positive versus -negative FMT may be the best way to resolve this question, especially in the context of IBS-focused clinical trials. I recommend that the questionable value of inclusion of testing for these ‘parasites’ into the donor screening protocol should be somehow included in the abstract, given that this might be the only part of the paper read by many.

We thank the reviewer for this comment. We have discussed this matter among the co-authors and decided to add a sentence on this subject to the abstract. However, we made a distinction between protozoa Dientamoeba Fragilis and Blastocystis spp. on this statement after elaborate discussion with the parasitology expert of our center, Dr. T. van Gool. Data on pathogenicity of Dientamoeba Fragilis is widely available and several international guidelines advise to treat carriership with Dientamoeba when gastro-intestinal symptoms are present and no other cause is found. To advice to stop screening for Dientamoeba in donor feces is in our opinion not supported by (our) data.

2. While the donor screening and testing protocol described is reasonably rigorous, I think the discussion should consider additional costs that they could be facing if they had to adhere to even stricter regulations:
   a. One potential pathogen that is already written in stone in the US by the FDA is enteropathogenic E. Coli (EPEC), which is further mandated to be tested for using nucleic acid amplification. EPEC is commonly found in healthy individuals, may a self-limited diarrheal illness, although little is known about the pathogenicity of its various strains. If it were included in the screening protocol of this group, they would have an even higher rate of exclusion. Absence of EPEC testing should be noted in the discussion, as it is a serious burden for the donor programs in the US (and could become so in other countries as the regulators look to each other for else they should be doing).

We thank the reviewer for raising this matter. We have added the difference with regard to screening for EPEC between FMT centers in the discussion on page 18. We have now highlighted that if EPEC was included in our screening protocol, this would result in even higher rates of donor exclusion. Moreover, we elaborate on this matter in the discussion on page 21 by focusing on potential rising donor screening costs.

b. The investigators describe the logistic difficulties in procuring stool during the COVID era because their donors had to bring in donations from home. While COVID has certainly complicated operations of stool donor programs, consider that the FDA requires US donors to produce stool on site in a supervised bathroom to ensure chain of custody.

We agree with the reviewer that we could have elaborated a bit more on the method of sample collection. We adjusted the methods of stool sample collection on page 7. We believe that by using universal clean plastic containers for collecting stool the risk of transmitting communicable disease is negligible. We discuss the potential of rising donor screening costs when stool production in a supervised bathroom becomes mandatory in the discussion on page 21.

c. The enteric pathogen testing protocol uses 60-day brackets. However, the rate of transiently positive tests would likely be higher if every donation was required to be tested for all pathogens. The added costs obviously include the additional labs expenses. However, flickering positive results lead to increased rates of donor disqualification.

We thank the reviewer for this suggestion and adjusted the discussion on page 18 accordingly.

d. Excessive testing may not be limited to enteric pathogens. For example, some FMT donor protocols in the US require anti-nuclear antibody testing, as a potential biomarker of future autoimmunity risk.

We thank the reviewer for this remark and added testing for anti-nuclear antibodies on page 20 as an example of differences (or possible future adaptations) to our screening protocol that will increase screening costs

3. The authors should discuss their approach to discussing meaningless positive tests with their donors. Are donors notified of their test results and counseled? How do they deal with the potential anxiety that is then generated? The donors may think that they have some problem brewing or that they could transmit an infection to someone around them. If they do not have this discussion, why not?

We thank the reviewer for these relevant questions. Our IRB approved protocols stipulate to notify donors of any positive test results and counsel them accordingly. In our experience and to the best of our knowledge, these counselling sessions did not lead to anxiety in our potential donors. We also discuss the chance of finding clinically irrelevant test results beforehand in the informed consent progress.

In the manuscript (in the Methods section, sentence 165,166,167 we added our approach with discussing positive test values with our donors.

4. The authors state in the introduction that stool testing for SARS-CoV-2 is required. Unfortunately, in the US stool testing for SARS-CoV-2 is not FDA approved. However, frequent nasopharyngeal testing on donors is allowed in some programs. I suggest the authors remove ‘stool’ from their statement – the methods of testing for SARS-CoV-2 are not a universally settled issue at this time.

We have removed ‘stool’ in the statement in the introduction on page 4.

5. The discussion should make a point that high rates of donor exclusion in a typical FMT donor program illustrate the dangers of do-it-yourself (DIY) protocols and the advantages of a centralized ‘stool-bank’ model. Yet, flippant over-regulation can drive such models out of existence and encourage greater uptake of DIY practices and medical tourism.

We agree with the reviewer that we should make a statement on the dangers of do-it-yourself FMT. We added this to the Discussion on page 20-21 and Conclusion.

6. On the other hand, some commercial developers and academics argue that FMTs should go out of existence and be replaced with synthetic microbial products or alcohol-treated microbiota. However, these assertions are made without data that include microbiome-based endpoints. FMT has been shown to decrease the burden of antibiotic resistance genes in patients with recurrent C. difficile infections. Similarly, Sam Nooji et al. (Gastroenterology, 2021), showed that the prevalence of pks+ E. coli was either reduced or unchanged following FMT. How would synthetic products do with respect to these endpoints? I think the authors should anticipate these topics in their discussion because there is high likelihood that certain commercial players will capitalize on the ‘dangers’ of FMT suggested here (without evidence of harm in patients) and tout unvalidated advantages of their products.

We thank the reviewer for sharing his/her concern that there is a likelihood that commercial developers will capitalize on the dangers of conventional FMT, derived from our presented data. We added the development of synthetic microbial products in the discussion on page 22. We highlight the limitations of the use of synthetic microbial products, namely: 1) current synthetic microbial products comprised of limited strains poorly represent the complete gut microbiome and 2) data on transmission of uncovered potentially procarcinogenic or pathogenic species cannot be derived retrospectively from trials using synthetic products, 3) the absence of clinical data on the efficacy and long-term safety of synthetic microbial products.

Reviewer: Jeremiah Faith

The manuscript by Bernard et al., on the costs and screening challenges for FMT donors is an excellent resource that will be of interest for groups with their own FMT banks. Even more so, it is a great set of data and insights for laboratories/institutions looking to establish their own FMT banks. I have no major concerns. The major challenge with the read is that procedures very from bank to bank and regulations can vary across countries (and are not that specific anywhere to begin with). However this caveat is clearly stated and references to relevant additional reading are provided.

Response to the reviewers’ queries:

1. I think the labels on top of supplemental Tables 1 and 2 are switched.
2. Line 131 “existed” I think that should be “consisted” of non-smoking adults…
3. Line 160 “alfa” should be “alpha”

These points have been adjusted in the manuscript.

4. It would be nice to hear more opinions from the authors one which of the tests they think should and should not be included (the D. fragilis and Blastocystis was helpful in this regard, more along these lines would be even better)

This comment is in line with the request of reviewer 2; we added our opinion with regard to screening for enteropathogenic E. coli (EPEC) discussion on page 18. To the best of our knowledge, there is no additional data to support the decision to include or exclude pathogens in our current screening protocol. Therefore, we did not further elaborate on this matter.
