# Supplementary material for: Challenges and costs of donor screening for fecal microbiota transplantations
Source: PLoS One. 2022 Oct 20;17(10):e0276323. doi: 10.1371/journal.pone.0276323 (PMC9584411; doi:10.1371/journal.pone.0276323)
Supplement: Attachment — Submitted filename: Response_to_Reviewers_2_29SEP2022.docx [file pone.0276323.s004.docx]

Franck Carbonero, PhD

Editorial board PLOS ONE

Amsterdam, the Netherlands, September 28^th^, 2022

Re:

Manuscript title:	“Challenges and costs of donor screening for fecal microbiota transplantations”

Authors:	Mèlanie Bénard, Clara Bruijn, Aline Fenneman, Koen Wortelboer, Judith Zeevenhoven, Bente Rethans, Hilde Herrema, Tom van Gool, Max Nieuwdorp, Marc Benninga, Cyriel Ponsioen

Manuscript ID:	PONE-D-22-15731

Dear Dr. Carbonero,

Thank you very much for considering our paper entitled: “Challenges and costs of donor screening for fecal microbiota transplantations” for publication. We carefully addressed the additional comments of reviewer 2 and have made changes in the manuscript according to the suggestions. Please find our point-by-point answers below. All changes in the revised version of the manuscript are marked with the use of track changes.

We hope that the revised version of the manuscript fulfils the necessary criteria to be accepted.

We look forward to hearing from you.

Kind regards,

On behalf of the co-authors,

Cyriel Y. Ponsioen MD, PhD

Department of Gastroenterology & Hepatology

Amsterdam University Medical Centers

Amsterdam, The Netherlands

c.y.ponsioen@amsterdamumc.nl

The Netherlands

Tel: +31205662906

REVIEWERS COMMENTS

Reviewer: 2

Response to the reviewers’ queries:

The authors have provided thoughtful answers to the critiques and have done an admirable job overall.

We thank reviewer #2 for this kind overall comment.

I have two remaining comments, which need to be addressed:
1. The authors said they addressed the issue of supervised bathroom donations on page 21. I saw a table on page 21 with no discussion of this issue. A supervised bathroom is mandatory in manufacturing of FMT products in FDA-approved protocols. The cost should be acknowledged, including (1) logistics of recruitment of donors required to come to a facility and provide stool on demand; (2) facility fees for reserving a dedicated bathroom; (3) having a donor coordinator on site to receive the donations.
We thank the reviewer for this remark. We apologize for the confusion: in the previous version of the manuscript (“Revised Manuscript with Track Changes_5SEP2022”) we did not discuss the potential of rising donor screening costs when stool production in a supervised bathroom becomes mandatory in the discussion on page 21, but on page 20 (line 372-373).

We agree with reviewer #2 that stricter regulations can lead to higher associated donor screening costs. Therefore, in the new version we elaborate more on this issue. We are aware that the classification of human stool intended for FMT is under debate. Moreover, codes and regulations around the procurement of FMT (such as a dedicated bathroom) and associated screening costs differ between countries, depending on the classification used (e.g. medicinal product, biological product, human cell/tissue product, or substance of human origin). Scheeler (2019) and Merrick and colleagues (2020) discuss this matter extensively. These references have been added in the new version of the manuscript. In addition, we added lines 368-371 in the discussion on page 20 accordingly (“Revised Manuscript with Track Changes_28SEP2022”).

We also acknowledge that higher logistic demands on stool donation could result in higher costs. In our cohort, logistic problems resulted already in the loss of 35 donors during prescreening and 5 donors during screening. We described that logistic burdens are a deterrent for donor motivation in the discussion (lines 273 – 278). In the current version we specified the potential barrier of extra time and transportation problems around screening and stool donation. Higher demands on donors complicates recruitment and may result in higher drop-out rates.

Moreover, we highlighted that mandatory donation of feces in a dedicated supervised bathroom results in higher logistic, facility and employee expenses in lines 376-377.

References

Scheeler A. Where Stool is a Drug: International Approaches to Regulating the use of Fecal Microbiota for Transplantation. J Law Med Ethics. 2019;47(4):524-40.

Merrick B, Allen L, Masirah MZN, Forbes B, Shawcross DL, Goldenberg SD. Regulation, risk and safety of Faecal Microbiota Transplant. Infect Prev Pract. 2020;2(3):100069.

2. I did not see a mention of whether the donors are paid or not, and how much. This is an important issue. Commercial manufacturers of FMT-like products, e.g., Seres Health, Ferring Pharmaceuticals (Rebiotix) pay quite handsomely to their donors, e.g., ≥ $1,200 per month. This practice obviously introduces potential hazards, which are amplified hugely by not having a supervised bathroom.

We kindly point the reviewer to our methods on page 6 (line 125-127) in which we described the financial compensation for donors in our clinical FMT trials. We agree with reviewer #2 that high reimbursements for donors could induce risks by attracting potential fecal donors with undesirable motives. We would like to highlight that in our cohort no financial compensation (no donation fees and no travel expenses) was offered during the screening phase. The screening procedure existed of at least one physical appointment in the hospital to sign informed consent. Depending on the results of the extensive questionnaire, stool samples -collected in a plastic stool container- were handed in and screened during a second appointment. If applicable, routine biochemical analysis of blood was performed during a third visit. Only once qualified as fecal donors, financial compensation was offered with reimbursements ranging between €10 – 50 per donation plus additional travel expenses (e.g. travels to a facility to provide stool sample and/or undergo rescreening). We believe that in this way potential healthy fecal donors with questionable incentives will not be interested in participation in the trial(s). Moreover, we believe that by using universal clean plastic containers for collecting stool (handed out during the first appointment) the risk of transmitting communicable disease is negligible. We have added the sentence that no compensation was offered during the screening phase to the methods (page 6, line 125-127).
